# Human intracranial signal stability tracks anatomical accuracy after electrode reimplantation

**DOI:** 10.1088/1741-2552/ae805e

**Published:** 2026-07-14

**Authors:** Ryan B Leriche, Thomas A Wozny, Jeremy Saal, Julian C Motzkin, Kristin K Sellers, Philip A Starr, Edward F Chang, Prasad Shirvalkar

**Affiliations:** 1 Medical Scientist Training Program, University of Minnesota, Minneapolis, MN, United States of America; 2 Department of Neurological Surgery, University of California, San Francisco, San Francisco, CA, United States of America; 3 Weill Institute for Neurosciences, University of California, San Francisco, San Francisco, CA, United States of America; 4 Department of Neurology, University of California, San Francisco, San Francisco, CA, United States of America; 5 Department of Anesthesiology (Division of Pain Management), University of California, San Francisco, San Francisco, CA, United States of America

**Keywords:** deep brain stimulation, stereoelectroencephalography, neuropsychiatric disorders, personalized medicine, functional neurosurgery

## Abstract

*Objective.* Deep brain stimulation (DBS) for neuropsychiatric disorders increasingly relies on patient personalized approaches. One strategy to identify viable targets for personalized stimulation and biomarker detection uses a temporary, inpatient stereoelectroencephalography (sEEG) trial to identify optimal targets for permanent re-implantation. However, this approach reasons that neural signals are stable between repeated intracranial electrode implants, which is not yet validated. *Approach.* We characterize the spectral stability of local field potentials after electrode reimplantation from an ongoing single-center clinical trial using two-staged sEEG/DBS for chronic pain (NCT04144972). *Main results.* Repeat neurosurgical implant of intracranial electrodes reliably targeted intended trajectories across staged surgeries occurring months apart (2.1 ± 1.2 mm Euclidean distance across 17 brain targets, 5 participants). Anatomical targeting error was correlated with differences in recorded electrophysiological power spectra between stages (*r*(15) = 0.51, *p* = 0.03). This correlation was stronger for the subset of regions re-targeted exclusively for biomarker sensing (*r*(6) = 0.73, *p* = 0.04), and non-significant for therapeutic stimulation sites (*r*(7) = .23, *p* = 0.76). Similarly, a multiple regression model significantly predicted spectral distance (*F*(4, 13) = 5.99, *p* = 0.008, adjusted *r* = 0.70) with an interaction between electrode type and Euclidean distance (*t*(13) = 2.39, *p* = 0.03). *Significance.* Chronic intracranial EEG (iEEG) signals recapitulated temporary recordings in 17 of 17 brain regions with the major spectral peak within 1 Hz. Our results demonstrate the temporal stability of neural signals across months and validate the use of a two-staged iEEG approach for chronic DBS sensing in humans.

## 1. Introduction

Deep brain stimulation (DBS) is standard of care for medication refractory movement disorders and has demonstrated experimental promise as treatment for neuropsychiatric disorders [1]. While initial results for DBS of complex neuropsychiatric disorders were promising, blinded trials have had mixed outcomes [2–4]. Closed-loop DBS which provides therapeutic stimulation in response to symptom-relevant biomarkers may provide superior benefit to open-loop, particularly for these disorders [5]. This strategy has successfully treated the motor fluctuations in Parkinson’s disease and sporadic seizures of epilepsy [6, 7]. For complex neuropsychiatric disorders, optimal targets to deliver stimulation and record symptom-related electrophysiology are not known [5].

Personalizing brain targets could improve symptom-decoding accuracy, but per patient data-driven approaches are still under development [5, 8]. For chronic pain we used two-staged intracranial EEG (iEEG) to identify permanent DBS targets from 10–16 candidate regions during an inpatient trial period [8, 9]. First, we implanted temporary iEEG electrodes in brain regions hypothesized to be involved in chronic pain. Over the 10 d inpatient trial, we serially tested brain regions to find pain-relieving and pain-decoding sites empirically derived per participant. Second, we reimplanted optimal sites for permanent DBS with ambulatory iEEG, under the assumption that we could detect similar neural signals over time. This technical assumption has not yet been validated.

Here we report that staged iEEG reliably targets within-participant brain regions with stable neurophysiology between stages. First, we colocalized electrode locations between stages within region to identify spatially homologous neural sensing electrodes, referred to as *sense homologs*. Second, within sense homologs we found that the electrophysiological similarity was explained by implant accuracy. We demonstrate that despite the short-term nature of inpatient iEEG, it can reliably predict neural signals recorded from permanently implanted devices. Together, with our previous work showing that day-to-day variation in chronic pain levels can be predicted from spectral power bands of ambulatory iEEG [10], this provides empirical groundwork for future studies to assess how staged iEEG could inform personalized closed-loop DBS for chronic pain, and other complex neuropsychiatric disorders [8, 9, 11].

## 2. Materials and methods

### 2.1. Participants

Five participants with treatment-refractory chronic neuropathic pain (3 female/2 male, age range: 51–67 years) underwent staged iEEG implants as part of a clinical trial (NCT04144972). Participants were recruited from either the University of California San Francisco Center for Pain Medicine or referred by physicians in the United States. Written informed consent was obtained prior to any study involvement in accordance with the Declaration of Helsinki and the University of California San Francisco Human Research Protection Program.

### 2.2. Staged iEEG electrode implant by stereotaxic brain surgery

Participants were admitted to the hospital for a 10 d inpatient iEEG trial period involving implant of up to 9 stereoelectroencephalography (sEEG) depth electrodes each. Bilateral brain targets included: subgenual, anterior and middle cingulate cortices, basal ganglia, thalamus, periaqueductal gray, insula, and sensorimotor cortices. We performed preoperative stereotactic trajectory planning with Brainlab Elements software Version 2.5 (Munich, Germany) using T1 weighted MRI with contrast for anatomical localization. sEEG electrodes were implanted as previously described [12]. Briefly, we used a CRW stereotactic frame in ‘mohawk’ position and fiducial rods imaged by intraoperative O arm scan to co-register to preoperative MRI in Brainlab. Up to nine inpatient iEEG depth electrodes were implanted per participant (Ad-Tech SD08R-SD14R or PMT Corporation 2102-16-091). Inpatient iEEG depth electrodes contained 8–14 cylindrical electrodes of 0.8–1.1 mm diameter, 2–2.41 mm length and 3.5–5 mm pitch (table 1). Each electrode was stereotactically implanted with a twist drill and secured with skull-mounted anchor bolts. Externalized electrodes were connected to a 256-channel Nihon Kohden clinical neurophysiology recording and stimulation system (Model EEG-1200). A portable CT scan confirmed electrode locations intraoperatively. Based on inpatient brain stimulation and recording, four brain regions were re-targeted for permanent DBS implant: one for stimulation and one for sensing in each hemisphere [9, 13].

**Table 1.** Inpatient and ambulatory iEEG physical and recording specifications.

	Inpatient iEEG^a^	Ambulatory iEEG^b^
Physical		
Manufacturer	Ad Tech.	Medtronic
Lead model	SD08R-SP05X-000 SD12R-SP05X-000 SD14R-SP05X-000	3387
Contact diameter	1.1 mm	1.27 mm
Contact length	2.41 mm	1.5 mm
Contact pitch (center to center)	5 mm	3 mm
		
Recording		
Sampling rate	2000 Hz	250 Hz
Referencing scheme	Any (bipolar used)	Only bipolar available

a For P2, PMT Model 2102-16-091 depth iEEG lead was used (0.8 mm contact diameter, 2 mm length, and 3.5 mm pitch).

b One of P4’s right hemisphere electrodes used Medtronic’s Model 3391 deep brain stimulation lead (contact diameter 1.27 mm, contact length 3 mm, and 7 mm contact pitch); because of its different dimensions this electrode was excluded for analysis.

Abbreviations: intracranial electroencephalography (iEEG).

Following inpatient iEEG, we then implanted ambulatory iEEG depth electrodes (Medtronic 3387) targeting a subset of regions that were previously sampled with the inpatient iEEG electrodes. The time between inpatient iEEG explant and ambulatory iEEG implant was 45.6 ± 27.0 d (mean ± standard deviation) with a range of 19–88 d. Participants underwent repeat MRI and CT Brain for target localization. Images were imported into StealthStation™ Surgical Navigation System Version 8 (Medtronic, USA) for trajectory planning and fused with the previous implant intraoperative CT. Under general anesthesia, we used a Leksell frame to guide stereotactic implant of four cylindrical quadripolar Medtronic DBS leads (Model 3387) with electrodes of 1.27 mm diameter, 1.5 mm length, and 3 mm pitch (table 1). Two leads per hemisphere were connected to 60 cm lead extenders (Medtronic model 37087) subcutaneously tunneled to bilateral internal pulse generators (Medtronic Summit RC + S DBS[14]) implanted in the infraclavicular space. Electrode integrity was confirmed with normal range bipolar impedance values (i.e. <3000 ohms). The investigating neurologist and neurosurgeon confirmed electrode location from a postoperative high-resolution CT scan two months later.

### 2.3. iEEG electrode localization and Euclidean distance error

For each participant, we used the Statistical Parametric Mapping toolbox ver.12 [15] in MATLAB Version 2023b (MathWorks, USA) to co-register the ambulatory to inpatient stage CT scan using a rigid body, linear affine transformation, and a mutual information maximization algorithm before reslicing into a common space with a 4th degree B-spline interpolation. Then, 3D Slicer software [16] was used to create a volumetric 3D mask for each electrode contact artifact using a combination of intensity thresholding and manual mask refinement. These segmentation masks were imported into MATLAB for further analysis. Electrode contact locations were defined as the geometric center of each electrode contact mask in patient native space.

With ambulatory and inpatient iEEG electrodes both in patient native space, spatially homologous electrodes were found from each stage, so called sense homologs, by finding the nearest bipolar pairs. Since ambulatory iEEG could only record from a bipolar montage, we aligned by bipolar pairs rather than by individual contacts for consistency with our electrophysiological analyses. Specifically, the geometric centers of each bipolar pair were found by averaging the 3D coordinates of the two contacts. We then found the Euclidean distance between the theoretical ambulatory and inpatient bipolar channel locations:

\begin{align*}{\text{Euclidean distance}} = \sqrt {{{\left( {{x_{{\mathrm{ambulatory}}}} - {x_{{\mathrm{inpatient}}}}} \right)}^2} + {{\left( {{y_{{\mathrm{ambulatory}}}} - {y_{{\mathrm{inpatient}}}}} \right)}^2} + {{\left( {{z_{{\mathrm{ambulatory}}}} - {z_{{\mathrm{inpatient}}}}} \right)}^2}} .\end{align*}

Per brain region, the inpatient and ambulatory bipolar pairs with the minimum Euclidean distance were defined as sense homologs.

Note that we defined inpatient iEEG bipolar electrodes to be adjacent (e.g. 1 and 2), but ambulatory iEEG electrodes to have non-consecutive contacts (e.g. 1 and 3). This was done to better approximate the spacing of bipolar recordings between stages since the inpatient iEEG contact center-to-center spacing was larger than the ambulatory iEEG contacts (table 1).

### 2.4. Local field potentials (LFP) acquisition, processing and analysis

Inpatient iEEG signals acquired with the Nihon Kohden clinical electrophysiology system were sampled at 2–10 kHz, referenced to a deep contact in white matter, down sampled to 1 kHz for and bipolar referenced for analysis [17]. Ambulatory iEEG signals were recorded using the Medtronic Summit RC + S [14] over radiofrequency to Bluetooth streaming sampled at 250 or 500 Hz, after onboard bandpass filtered from 10–125 Hz, and using a bipolar montage with up to 2 channels per electrode.

We used clean LFP recordings from both stages that occurred at least 1 min after any stimulation [18]. After notch filtering at 60 Hz, power spectra for each recording were computed with multitaper spectral decomposition (3 Slepian tapers, 1–125 Hz in 0.5 Hz steps) for inpatient iEEG in Python with the MNE [19] package and the ambulatory iEEG in MATLAB with the FieldTrip [20] toolbox. Noisy spectra were removed by manual inspection. To ensure that pain symptoms differences did not influence spectral composition, we selected recordings matched for pain intensity between stages, using recordings associated with the most common numeric pain rating value across stages (e.g. recordings associated with a pain intensity report of 4/10). After preprocessing, there was a range of 20–159 inpatient recordings lasting 20 s and 13–99 ambulatory recordings each lasting 10–30 s, across participants (table 2). The ambulatory stage used shorter recordings because of the well-documented packet-loss inherent to the Medtronic Summit RC + S system[21].

**Table 2.** Comparison of peak frequencies between inpatient and ambulatory stages.

Regions	Inpatient peakfrequency	Ambulatory peak frequency	Change peakfrequency	Equivalence testing *p*-values	
				Left tail	Right tail
P1 | left caudate	8.9 ± 0.5 Hz (*N* = 20)	8.6 ± 0.6 Hz (*N* = 18)	0.36 Hz [0.00, 0.72 Hz]	9.2 × 10^−09^	7.9 × 10^−04^
P1 | left anterior cingulate cortex	8.5 ± 0.8 Hz (*N* = 37)	8.3 ± 0.9 Hz (*N* = 24)	0.16 Hz [−0.26, 0.58 Hz]	1.3 × 10^−06^	1.8 × 10^−04^
P1 | right thalamus	13.4 ± 0.6 Hz (*N* = 33)	13.4 ± 0.7 Hz (*N* = 13)	−0.01 Hz [−0.42, 0.40 Hz]	1.8 × 10^−05^	1.3 × 10^−05^
P1 | right anterior cingulate cortex	9.3 ± 1.0 Hz (*N* = 32)	9.2 ± 0.9 Hz (*N* = 20)	0.11 Hz [−0.45, 0.66 Hz]	1.9 × 10^−04^	1.4 × 10^−03^
P2 | right anterior cingulate cortex	7.3 ± 0.9 Hz (*N* = 25)	7.4 ± 0.9 Hz (*N* = 43)	−0.17 Hz [−0.62, 0.28 Hz]	3.4 × 10^−04^	2.3 × 10^−06^
P2 | right thalamus	21.3 ± 1.1 Hz (*N* = 26)	21.1 ± 1.3 Hz (*N* = 37)	0.12 Hz [−0.49, 0.73 Hz]	3.7 × 10^−04^	3.1 × 10^−03^
P3 | left globus pallidus internus	9.0 ± 0.5 Hz (*N* = 25)	9.1 ± 0.5 Hz (*N* = 52)	−0.14 Hz [−0.36, 0.09 Hz]	1.5 × 10^−10^	6.3 × 10^−15^
P3 | left subgenual cingulate cortex	9.2 ± 0.9 Hz (*N* = 27)	9.4 ± 1.0 Hz (*N* = 88)	−0.20 Hz [−0.64, 0.23 Hz]	3.3 × 10^−04^	3.2 × 10^−07^
P3 | right thalamus	6.7 ± 0.8 Hz (*N* = 29)	6.4 ± 0.5 Hz (*N* = 99)	0.23 Hz [−0.01, 0.47 Hz]	0.0E + 00	5.8 × 10^−09^
P3 | right subgenual cingulate cortex	9.4 ± 1.0 Hz (*N* = 27)	9.7 ± 0.9 Hz (*N* = 88)	−0.34 Hz [−0.74, 0.06 Hz]	9.6 × 10^−04^	2.9 × 10^−09^
P4 | left caudate	9.2 ± 0.9 Hz (*N* = 53)	8.8 ± 0.7 Hz (*N* = 49)	0.48 Hz [0.17, 0.79 Hz]	6.6 × 10^−15^	8.8 × 10^−04^
P4 | left anterior cingulate cortex	7.7 ± 1.0 Hz (*N* = 66)	7.2 ± 0.7 Hz (*N* = 50)	0.56 Hz [0.23, 0.89 Hz]	3.8 × 10^−15^	5.1 × 10^−03^
P4 | right thalamus	6.2 ± 0.9 Hz (*N* = 58)	6.6 ± 0.6 Hz (*N* = 40)	−0.33 Hz [−0.64, −0.03 Hz]	2.9 × 10^−05^	3.1 × 10^−13^
P5 | left anterior cingulate cortex	7.5 ± 0.6 Hz (*N* = 159)	7.3 ± 0.6 Hz (*N* = 32)	0.25 Hz [0.01, 0.49 Hz]	0.0E + 00	5.0 × 10^−09^
P5 | left caudate	7.6 ± 0.4 Hz (*N* = 148)	7.3 ± 0.4 Hz (*N* = 18)	0.26 Hz [0.05, 0.46 Hz]	0.0E + 00	8.0 × 10^−11^
P5 | right thalamus	9.2 ± 0.5 Hz (*N* = 156)	9.5 ± 0.6 Hz (*N* = 32)	−0.22 Hz [−0.43, −0.01 Hz]	8.5 × 10^−12^	4.8 × 10^−23^
P5 | right superior frontal gyrus	7.2 ± 0.5 Hz (*N* = 159)	6.7 ± 0.6 Hz (*N* = 29)	0.52 Hz [0.32, 0.71 Hz]	0.0E + 00	1.5 × 10^−06^

Abbreviations: Participant (*P*).

Spectra were parametrized into periodic and aperiodic power components with the ‘fitting oscillations & one over f’ or FOOOF algorithm version 1.0.0 [22]. FOOOF settings were as follows: frequency range 2–35 Hz, peak width range 2–5 Hz, max number of peaks 4, min peak height 0.1, peak threshold 2, aperiodic mode fixed. We reasoned that comparing the periodic power components between sense homologs would control for hardware and analogue processing differences between iEEG recording systems. First, we aimed to assess the signal stability at each bipolar contact pair across reimplant stages by calculating *spectral distance*. We computed spectral distance by taking the absolute difference between the mean inpatient and ambulatory periodic power component spectra per brain region. Second, we used the FOOOF algorithm on the grand averaged inpatient iEEG spectrum from each bipolar recording contact pair to identify the major spectral peak, defined here as the largest amplitude peak in the periodic component. For each inpatient iEEG major peak, we found the nearest ambulatory iEEG peak frequency. Notably, we restricted our analysis to the major spectral peak rather than pre-defined frequencies bands to account for regional differences in spectral activity. By testing if sense homologs shared their major spectral peak, we could assess the stability of staged iEEG.

### 2.5. Statistical analyses

Our statistical approach assessed if major spectral peaks from inpatient iEEG were recapitulated in ambulatory iEEG. Per region, we compared if the center peak frequency across sessions were similar between the ambulatory and inpatient stages by equivalency testing of two simultaneous one-sided tests [23]. The null hypothesis was that the mean inpatient major spectral peak differed by more than an equivalence interval from the mean ambulatory major spectral peak. We chose an equivalence interval of Δ = 1 Hz to match the frequency resolution of the spectra. The two simultaneous one-sided tests rejected two aspects of the null hypothesis: a right-sided test assessing if the difference between two means was greater than $\Delta $, and a left-sided test assessing if the difference between two means was less than $ - $. Since the two tests were repeated for each of the 17 regions (i.e. 34 tests), we accounted for the multiple-comparisons problem with the Benjamini & Hochberg false-discovery rate correction [24]. After correction if the *p*-values from both tests were less than or equal to alpha = 0.05, then the null hypothesis was rejected. We then accepted the alternative hypothesis that the major spectral peak between the inpatient and ambulatory stages was sufficiently close to be considered practically equivalent.

We then assessed the relationship between spectral distance and Euclidean distance for differing electrode types (i.e. all electrodes, biomarker sensing electrodes, and therapeutic stimulation electrodes). First, a Pearson’s correlation was used to identify linear relationships per electrode type (figure 2(B)). Second, a multiple regression was run to assess if spectral distance across regions was predicted by an interaction between Euclidean distance and electrode type (biomarker sensing or stimulation). In Wilcoxon notation this was spectral distance ∼ Euclidean distance * electrode type. Dummy variable coding was used with 1 for biomarker sensing electrodes and 0 for stimulation electrodes. An ANOVA tested if individual coefficients differed from zero with an alpha = 0.05 used to determine statistical significance. To assess model quality, the residuals were visualized and tested for being non-normal and heteroscedastic using the Jarque–Bera test [25],and Breusch–Pagan test [26],respectively (supplementary figure 1). Note that brain regions were treated as independent observations meaning that data were pooled across participants. Specifically, each participant only had 2–4 regions which was too few to model participant-level effects (table 2).

## 3. Results

### 3.1. Staged iEEG reliably retargeted the same individual’s brain region

**Figure 1.**
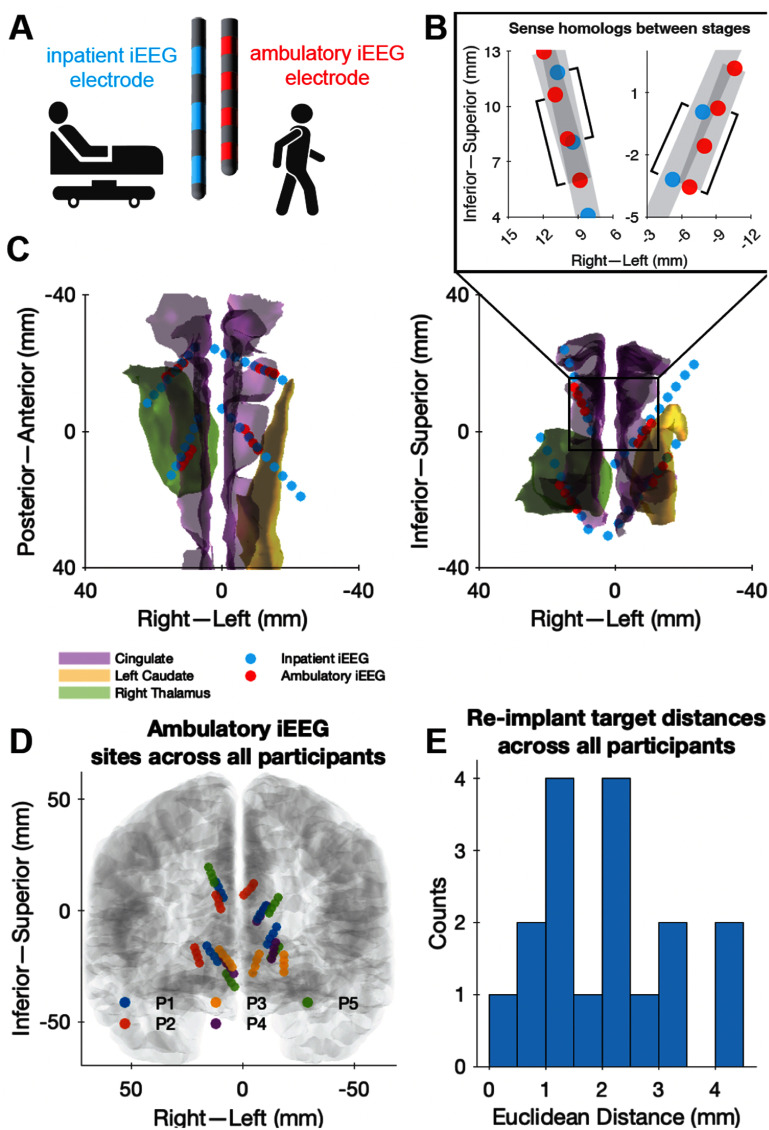
A staged iEEG paradigm reliably re-targets brain regions. (A) Schematic of inpatient iEEG followed by ambulatory implant. (B) Inset of figure 1(C) showing the nearest electrode pairs for P1 between the inpatient (light blue) and ambulatory (red) intracranial EEG stages. (C) For P1 staged iEEG electrodes were implanted within the cingulate cortices in purple, the left caudate in yellow and the right thalamus in green. (D) The ambulatory intracranial EEG implants for the entire cohort (P1–P5). (E) The distribution of Euclidean distance between the inpatient and ambulatory iEEG implant across brain regions. Abbreviations: intracranial electroencephalography (iEEG), Participant (*P*).

Inpatient iEEG electrode targets were accurately reimplanted with a different stereotactic system for ambulatory iEEG (figure 1(A)). To assess re-targeting accuracy, we identified sense homologs as the nearest electrode contact pairs between the inpatient and ambulatory iEEG (figure 1(B)). In P1 we implanted sense homologs within the bilateral anterior cingulate cortices, left caudate, and right thalamus (figure 1(C)). For all five participants, their inpatient iEEG motivated re-targeting for ambulatory iEEG sites within several regions: bilateral anterior cingulate cortices, left caudate, right thalamus, left globus pallidus internus, bilateral subgenual cingulate cortices, and the right superior frontal cortex (figure 1(D)). Across 17 brain regions (5 participants), the Euclidean distance between sense homologs was 2.1 ± 1.2 mm (0.42–4.41 mm) (figure 1(E)). Our staged iEEG reliably targeted diverse cortical and subcortical regions.

### 3.2. iEEG spectral similarity across stages correlates with anatomical targeting accuracy

**Figure 2.**
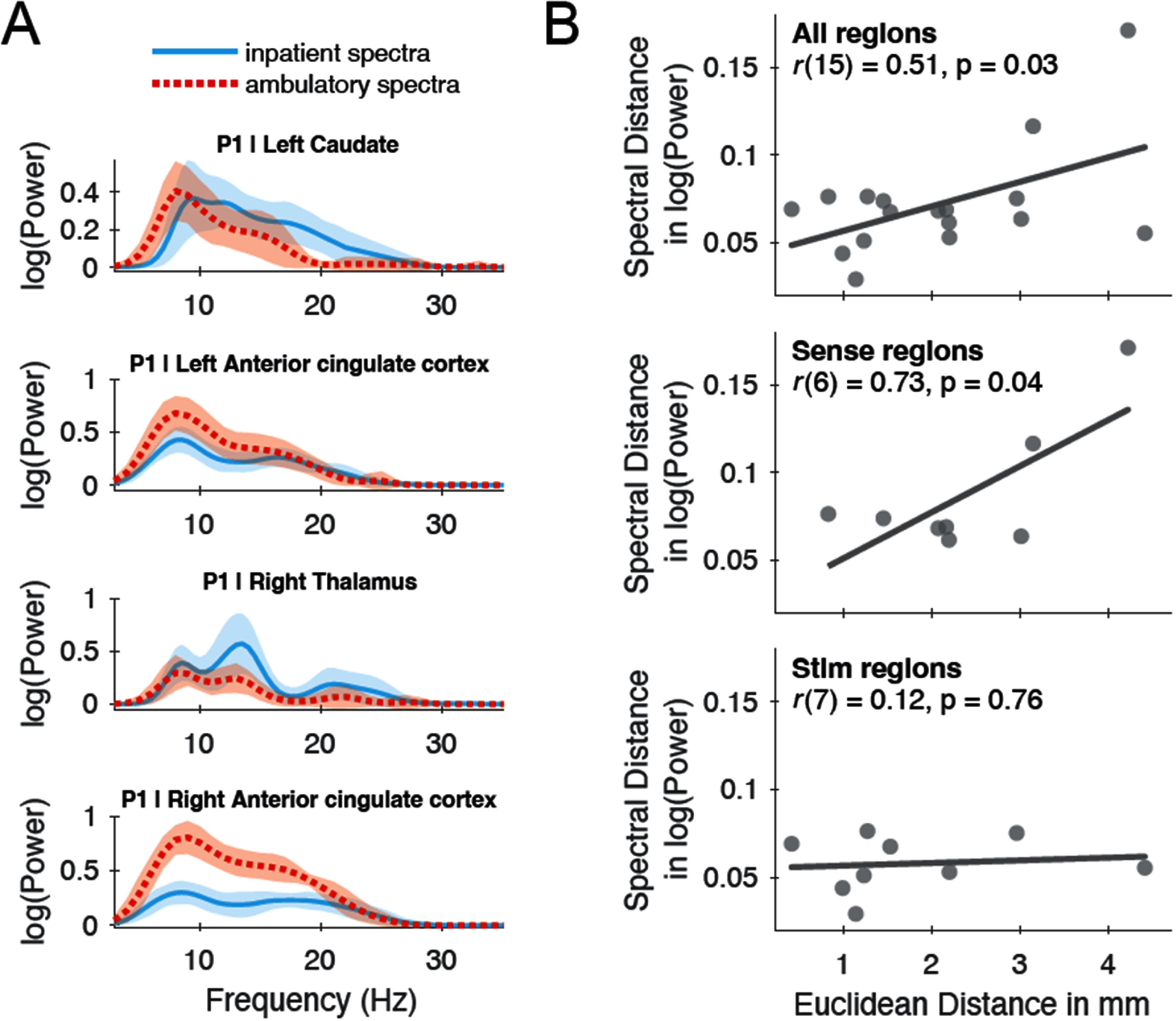
Between stages, iEEG spectra have similar shapes with spectral distance explained by Euclidean distance. (A) Example periodic component of neural power spectra from one participant (P1, see Methods for spectral parametrization) appear similar between the inpatient (light blue) and re-implanted ambulatory iEEG (dotted red) stages. Shading represents the standard deviation across sessions per stage. (B) The top panel shows Pearson’s correlation between Euclidean distance and spectral distance across all homologous neural sensing contacts between inpatient and ambulatory iEEG for 17 targeted regions from five participants. The lower panels show the same data, separated by sense homologs re-targeted for neural sensing only (middle panel), and those targeted for stimulation and sensing (lower panel). Linear best fit lines are shown for visualization with Pearson correlation coefficient with degrees of freedom and *p*-value per panel. Abbreviations: intracranial EEG (iEEG); Pearson correlation coefficient (*r*); Participant (*P*).

The periodic components of neural spectra recorded from the inpatient and ambulatory iEEG stages showed a similar shape within brain target (figure 2(A), supplementary figure 2(A) and (C), 3(A) and (C)). For sense homologs, spectral distance and Euclidean distance were positively correlated for all electrodes across participants (figure 2(B)) (*r*(15) = 0.51, *p* = 0.03). A subset of sense homolog contact pairs were used exclusively for biomarker sensing, while the remaining contact pairs were also used for therapeutic electrical stimulation. For the subset of electrodes re-targeted for biomarker sensing only, this correlation was even stronger (*r*(6) = 0.73, *p* = 0.04) while the correlation for electrodes re-targeted for therapeutic stimulation was non-significant (*r*(7) = 0.12, *p* = 0.76). This finding was further confirmed with a multiple regression model that significantly predicted spectral distance from Euclidean distance and electrode types (spectral distance ∼ Euclidean distance * electrode type) (*F*(4, 13) = 5.99, *p* = 0.008, adjusted *r* = 0.70). Ensuring the quality of model fit, the residuals did not appear heteroskedastic (supplementary figure 1(B)) nor did a Breusch–Pagan test find heteroskedasticity (*p* = 0.19). Further, the residuals appeared normal (supplementary figures 1(C)) and (A) Jarque–Bera test did not find deviation from normality (*p* = 0.50). Examining individual predictor variables showed a significant interaction between electrode type (sensing only vs stimulating only) and Euclidean distance (*t*(13) *=* 2.39, *p* = 0.03), but neither variable showed a significant main effect (electrode type: *t*(13)= − 1.22, *p* = 0.24; Euclidean distance: *t*(13)= 0.23, *p* = 0.82). This suggests that the spectral distance and Euclidean distance relationship held true for sense homolog contact pairs used for biomarker sensing, while electrical stimulation at distinct contact pairs was associated with a change in iEEG spectra stability. Overall, we found that differences in spectral content between ambulatory and inpatient iEEG signals increased with physical re-targeting distance, specifically for electrodes re-targeted for neural biomarkingsensing only.

### 3.3. Ambulatory iEEG signals recapitulated major spectral peaks from inpatient iEEG signals

**Figure 3.**
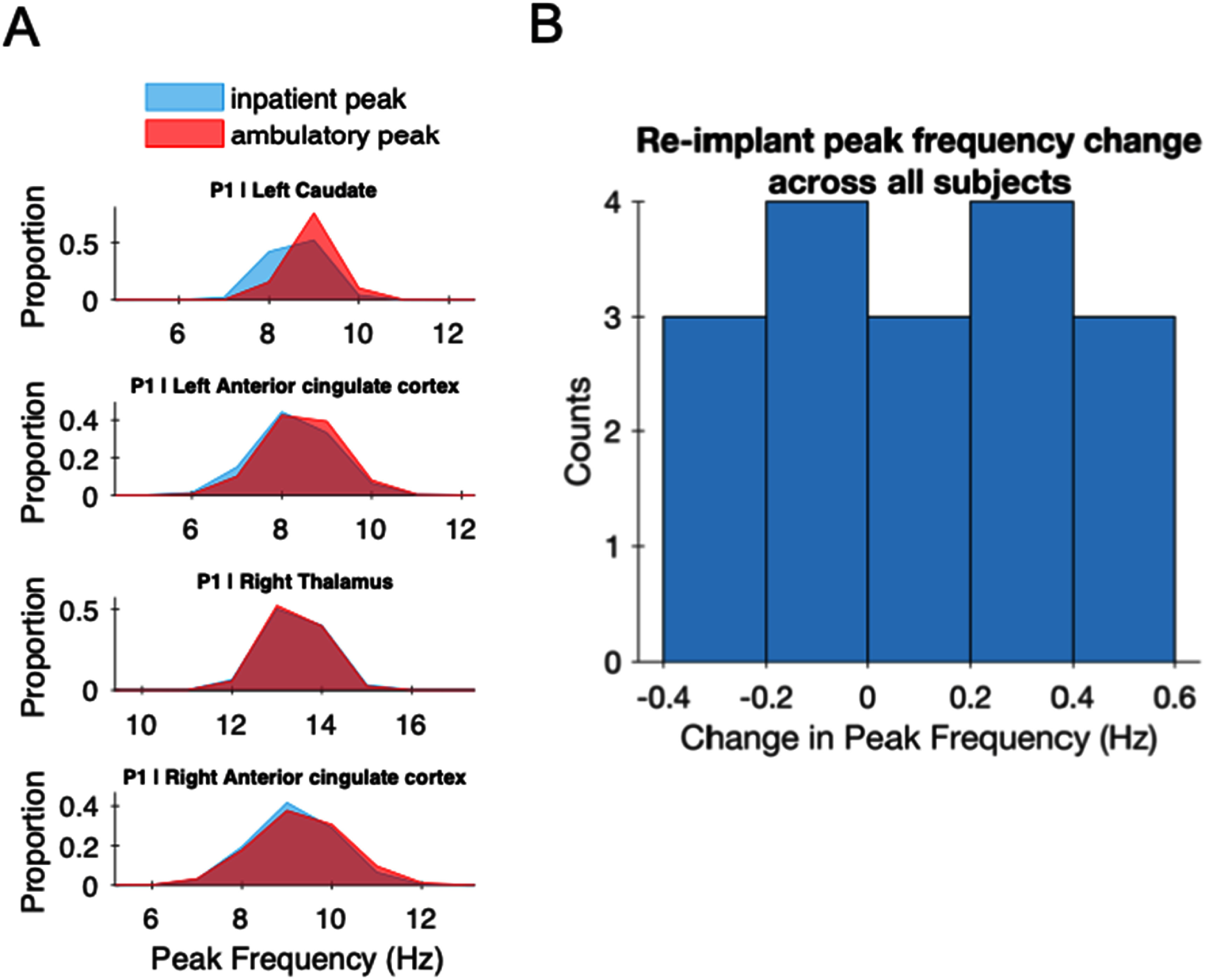
Sense homologs share the largest amplitude spectral peak. (A) For P1 the distributions of the largest amplitude peak frequencies between the inpatient and ambulatory iEEG stages. (B) A histogram of the change in mean peak frequency between inpatient and ambulatory iEEG across all brain regions. Note that all changes are within 1 Hz. Abbreviations: intracranial EEG (iEEG), Participant (*P*).

Major spectral peaks from the inpatient stage were reliably recaptured after reimplantation for the ambulatory stage (figure 3). For all 17 regions, equivalence testing showed that the largest amplitude peak frequency was within 1 Hz between stages (figure 3(B)). Table 2 provides descriptive statistics of the peak frequencies, and the right-sided and left-sided p-values for the mean difference in peak frequencies between inpatient iEEG and ambulatory iEEG. While the major spectral peak was consistent between stages (table 2, all left-sided *p*-values ⩽0.05 and right-sided *p*-values ⩽0.05), note that the shape of some neural spectra were less consistent (figure 2(A), supplementary figures 2–3).

## 4. Discussion

We characterized the anatomical distance and neural signal similarity from neurosurgical re-implantation between inpatient and ambulatory iEEG electrodes across 17 brain regions in five participants. We found that anatomical retargeting distance was associated with deviations in the expected iEEG spectral content. This suggests that inpatient iEEG recordings can guide permanent implant location, and signals in pain-relevant regions can maintain stability over time in a manner that can be recaptured with repeated electrode implantation.

### 4.1. Stability of human iEEG recordings

Although staged iEEG involves two penetrations of brain parenchyma for the inpatient and ambulatory stages, clinical and preclinical evidence supports the notion of signal stability. Staged iEEG is used in epilepsy to identify seizure foci for subsequent permanent neuromodulation [27]. In animals, adjustable microelectrode recordings are an established technique using similar repeated penetrations of white and gray matter up to once daily [28–30]. While signal stability from very fine diameter (<100 *µ*m) electrodes has been extensively studied in animals, we address the gap in how commercially available electrode re-implantation influences signal stability in humans.

Prior studies have reported mixed findings of iEEG biomarkers in complex neuropsychiatric disorders. In treatment resistant major depression, iEEG recordings from the subcallosal cingulate in the inpatient setting showed decreased beta power with euthymia [31]; however, a separate ambulatory cohort showed increased beta power with euthymia [32]. For obsessive-compulsive disorder, no consistent oscillatory biomarker in the ventral striatum has emerged [33–35]. Several explanations could explain these inconsistencies. Different surgical targeting between studies, even several millimeters, may result in varying symptom-related electrophysiology. Further, human iEEG symptom biomarkers may vary across timescales, be altered by DBS, and/or have unique profiles per subject [8, 36]. A staged iEEG approach to DBS can help clarify these possibilities and provide a reproducible technique for personalized biomarker identification.

Staged iEEG had accurate within-subject targeting and has precedence for symptom-decoding across timescales which could aid in the discovery of chronic pain neural biomarkers. We report within-participant accuracy (mean Euclidean distance 2.1 mm) comparable to a previous meta-analysis of actual to planned target site for frame-based systems (1.05–2.81 mm, 95% confidence interval) [37]. Previously, staged iEEG has been piloted in a *N*-of-1 study of treatment resistant depression in identifying a personalized ambulatory iEEG site [38]. Amygdala gamma power tracked depression between stages, and served as a stable input signal for closed-loop DBS over two months [38]. Here we similarly found that neural spectra exhibited stable peak frequencies between stages (table 2, figure 3), extending previous findings from a single region [38] to 17 regions. Together with a previous study from our team demonstrating that ambulatory iEEG can track chronic pain states [10], the present results suggest that temporary inpatient recordings could inform ambulatory iEEG sites for chronic pain decoding. Future work should examine the biomarker stability between inpatient and ambulatory iEEG over varying timescales. This may shed light on the electrophysiological mechanisms of immediate versus long-lasting DBS symptom relief.

Longitudinal signal stability may depend on whether a particular site was stimulated chronically. Spectral distance between inpatient and ambulatory iEEG recordings was predicted by and correlated with Euclidean distance for regions implanted exclusively for ambulatory DBS biomarker recording (i.e. no stimulation, figure 2(B)). While all recordings occurred with DBS off, it is possible that even chronic intermittent stimulation could cause lasting changes in local neural spectra. For example, in a prior study of four epilepsy patients turning off thalamic DBS was associated with gradual impedance increases for hours to days among stimulated contacts, but not unstimulated contacts [39]. Similarly, during breaks in continuous DBS for Parkinson’s disease, subthalamic beta oscillations were diminished between the initial programming session and at 1 year follow up, but only among stimulated subthalamic nucleus contacts [40]. Such impedance and LFP changes suggest distinct plasticity between stimulated and unstimulated neural populations. Considering local stimulation effects provides important context that will inform the development of longitudinal symptom biomarkers for complex neuropsychiatric disorders.

### 4.2. Hardware considerations for a staged iEEG approach

For a two-staged iEEG approach, using two surgical electrode implantation techniques for the inpatient stage (sEEG) and ambulatory stage (DBS) warrants careful surgical planning. Trajectories in the sEEG stage should be defined in anticipation of a future DBS trajectory, which may lead to slightly different burr hole locations than used for a single stage implant. This is because sEEG uses small-diameter, externalized, skull-mounted bolts whereas DBS requires burr holes to mount a subcutaneous electrode securement device, meaning more space is required between skull entry sites for DBS than for sEEG. While different trajectories through individual skull entry points may reach the same target, using the same burr holes for both stages reduces the total skull defects and may improve implant accuracy [41]. Further, DBS requires a large diameter metal cannula, which poses greater safely risks when passing through vascular areas compared to sEEG. This may preclude permanent DBS implant of regions close to sulcal vessels or eloquent cortex. In two-staged electrode implants, sEEG trajectory planning would ideally maximize safety and accuracy for subsequent DBS by planning overlapping skull entry points.

### 4.3. Alternatives to staged iEEG for personalized neuromodulation

Beyond staged iEEG, other personalized targeting methods are under study. In single-stage hybrid sEEG-DBS for depression, DBS targeting has been based on a holographic 3D anatomy visualization, rather than replication of sEEG signals [42]. A single stage hybrid sEEG-DBS approach can inform major depression circuit pathophysiology and DBS parameter selection [43, 44]. However, a staged iEEG approach could go further to theoretically identify novel targets for stimulation and biomarker discovery for various disorders [38]. Deep neuromodulation targets could also be personalized by potentially testing for acute effects with noninvasive methods such as low intensity focused ultrasound [45], although exact mechanisms of ultrasound stimulation remain unclear. Emerging work is also focused on using neuroimaging methods such as precision functional mapping (based on subject-level topographic variability from canonical functional networks) [46], or within-participant network targets defined from graph theory [36]. Neuroimaging based targets could be empirically assessed with staged iEEG, or guide the initial inpatient iEEG targeting based on a participant’s functional network.

### 4.4. Limitations

Our findings are limited to iEEG signals from 17 brain regions across 9 hemispheres from 5 participants. Similarly, our analysis of spectral distance to Euclidean distance (figure 2(B)) of only biomarker sensing or therapeutic stimulation electrodes was further limited to only 9 regions and 8 regions, respectively. We reasoned that periodic power would account for the different iEEG systems used between stages that have unique electrode geometries, recording montages, and analogue filtering steps (table 1). It is possible that these differences would prevent comparative analysis of iEEG signals. Regardless, we found stable neural signals between stages (figure 3). We focused on one inpatient iEEG system and ambulatory iEEG system for consistency in our small study, but larger studies should examine signal performance using electrode hardware from different manufacturers.

## 5. Conclusion

Human iEEG signals were recapitulated from diverse cortical and subcortical regions after electrode reimplantation. Our findings support the stability of iEEG signals within individuals between inpatient and ambulatory iEEG. Future work should examine the stability of symptom decoding across timescales and if inpatient iEEG signals could inform personalized closed-loop DBS for chronic pain.

## Data Availability

The data that support the findings of this study will be openly available following an embargo at the following URL/DOI: https://doi.org/10.5061/dryad.8931zcs3r [47]. Supplementary staged-iEEG Figures available at: https://doi.org/10.1088/1741-2552/ae805e/data1.
